# Designing a knowledge translation mentorship program to support the implementation of evidence-based innovations

**DOI:** 10.1186/s12913-015-0863-7

**Published:** 2015-05-14

**Authors:** Anna R. Gagliardi, Fiona Webster, Sharon E. Straus

**Affiliations:** University Health Network, Toronto, Canada; University of Toronto, Toronto, Canada; St. Michael’s Hospital, Toronto, Canada

**Keywords:** Mentors, Quality improvement, Qualitative research

## Abstract

**Background:**

Healthcare professionals require training in knowledge translation (KT) to implement evidence-based healthcare innovations. Mentorship is an effective training strategy that could be used to develop KT capacity but it has largely been used to train clinicians. The purpose of this study was to explore preferences for KT mentorship design.

**Methods:**

Interviews were conducted with 54 Canadian researchers and research users who varied by profession, department, career stage and sex. Participants were asked about KT needs, views on mentorship as a strategy to develop KT capacity, and suggestions for program design. Grounded theory technique and thematic analysis were used to collect and analyse data.

**Results:**

Participants uniformly expressed interest in mentorship over other forms of learning about KT because it would provide credible, tailored information when needed. A variety of options for program content, format and delivery were recommended, suggesting the need for flexibility according to KT needs. Leadership, infrastructure, culture change and incentives may also be needed to foster KT mentorship. Views were mixed on whether mentors should be KT experts or subject or clinical experts with KT experience, and embedded in, or external to organizations.

**Conclusions:**

These findings can be used to develop or evaluate KT mentoring programs. Further research is needed to evaluate different models in which the mentor may be an internal or external KT expert or subject expert with experience in KT, and establish the core curriculum of a training program specific to KT and how it could best be reinforced with mentoring.

**Electronic supplementary material:**

The online version of this article (doi:10.1186/s12913-015-0863-7) contains supplementary material, which is available to authorized users.

## Background

Healthcare organizations worldwide strive to optimize the organization, delivery and outcomes of services. However, there remains a need to improve the implementation and use of innovative knowledge, technology or practices as many patients do not receive high-quality care that is recommended by evidence-based guidelines [1, 2]. Multiple, contextual issues interact and contribute to these circumstances, including patient, provider, institutional, system, political, social and cultural factors [3]. Furthermore, many interventions commonly used to implement innovations, for example, educational materials and meetings, opinion leaders, or audit & feedback, are inconsistently effective, and require tailoring based on contextual issues to enhance their impact [4]. Despite these challenges, it appears that most health professionals are aware and accepting of evidence-based innovations, but struggle with how to implement them [5].

Knowledge translation (KT) refers to the practice of implementing innovations to improve patient outcomes and system performance [6]. KT encompasses a variety of approaches including dissemination (passive sharing of information about innovations through messages, presentations, or publications); implementation (active and purposeful promotion of innovations through a variety of educational, social, organizational, policy or incentive mechanisms); and integrated KT (collaboration among researchers and research users) [7]. KT is complementary to, but distinct from evidence-based medicine or practice which focuses on the formulation of clinical questions, acquisition and critical appraisal of information, and its use in clinical decision-making [8]. Considerable research involving researchers and research users in a range of settings and countries revealed limited KT knowledge and skill [9–13]. Therefore, it is imperative that strategies be developed that equip or support health professionals to more routinely and effectively implement innovations, including researchers who develop innovations, and research users (clinicians, managers, policy-makers) who apply them in policy or practice.

KT training programs have been established in the United States, United Kingdom and Canada [14–17]. Most of these featured a mixed design including didactic presentations, and in-person or remote interaction with instructors. Notably, those who participated in one program that largely consisted of group discussion recommended that the program incorporate one-on-one mentorship to reinforce learning [14]. While two other programs offered mentorship, details about program design were few [15, 17]. Mentorship is an interactive process meant to promote learning and development that is based on educational and social learning principles [18]. It has been applied in the corporate sector where senior organizational mentors help mentees develop professional and psychosocial skills [19, 20]. Mentees experienced more promotions, higher salaries, less stress, and greater career satisfaction than those who were not mentored [21, 22]. Mentorship has also been used to develop clinical skills among student or novice physicians and nurses, and offer academic career guidance to clinical and doctoral researchers [23–28]. In these healthcare contexts mentorship was associated with career promotion and satisfaction, as well as clinical performance and patient safety.

Mentorship is an effective approach for developing knowledge and skill that could potentially be used to enhance KT capacity among researchers and research users, either alone or in conjunction with KT training programs. However, no empirical research in healthcare settings has specifically examined its application for this purpose. Hence, we conducted a systematic review of management and social sciences literature to identify essential components of mentoring programs that could be adapted for KT mentorship [29]. Few studies were identified that specifically aimed to improve knowledge or skill as most focused on the development of psycho-social skills, or career progression, satisfaction or retention. Mentoring programs varied widely across studies so no conclusions could be drawn about the ideal format and content of KT mentoring. Having ascertained the paucity of available, relevant information that would inform the ideal components of a KT mentoring program, our overall aim was to explore how mentoring could be used to help healthcare professionals, including researchers and research users, implement evidence-based healthcare innovations. More specifically, the objective of this study was to interview researchers and research users to learn about their KT needs and preferences for the design of a KT mentorship program and, in so doing, generate insight on the infrastructure and capacity required to deliver KT mentorship.

## Methods

### Approach

Since little was known about KT mentorship preferences, a grounded theory approach was used to inductively derive needs, views and suggestions from the data rather than according to the restricted components of an established theory [30]. Semi-structured interviews were used to collect data. Rigour was optimized through purposive sampling, inductive analysis, examining deviant responses, and comparison of independently-derived themes, and complying with Relevance, Appropriateness, Transparency and Soundness (RATS) principles (Additional file 1) for the reporting of qualitative research [31, 32]. Ethical approval was obtained from the University Health Network in Toronto, Canada (09-0566-AE). Participants provided written informed consent prior to being interviewed.

### Sampling and recruitment

Participants were purposively identified from the University of Toronto faculty or departmental Internet sites. This environment provided access to researchers (doctoral, clinical) and research users (clinicians working in hospitals, and clinical and administrative managers in both healthcare and academic settings). Individuals were invited to participate by regular and electronic mail. A reminder email was sent to non-respondents at two and four weeks from initial contact. The intent was to recruit ten candidates from the Faculty of Nursing and each of three departments in the Faculty of Medicine who differed in non-mutually exclusive fashion by type of investigator (PhD, clinician), career stage (self-defined by participants as early, mid or late), and sex (male, female) for a minimum total of 40 participants. Detailed information from representative, rather than a large number of cases is needed in qualitative research [30]. To establish sample size, sampling was concurrent with data collection and analysis, and proceeded until no further unique themes emerged from successive interviews (saturation). This was determined by discussion of the coding scheme between two independent reviewers.

### Data collection

Interviews were conducted with consenting participants by a research associate who was trained by the principal investigator. Participants were asked to describe their awareness, knowledge and practice of KT; KT needs; preferences for developing KT knowledge or skills; prior experience with mentorship; suggestions for KT mentorship program design; and anticipated challenges associated with KT mentorship. The interview guide (Additional file 2) was modified following the first interview to merge two questions about current use of KT, and to shorten one question about KT needs. Mutual understanding of KT was established with all participants before asking about the relevance of mentorship. Telephone interviews of 30 to 45 min were audio-recorded, then transcribed verbatim by a professional transcriptionist. Interviews were conducted from September 2 to November 16, 2009.

### Data analysis

Unique themes were inductively identified through iterative stages [33, 34]. Transcripts were read to identify, define and organize themes. A codebook was developed to illustrate themes with exemplary quotes. Transcripts were reviewed (constant comparative technique) to assess whether and how to expand or merge thematic codes. Transcripts and the codebook were analyzed independently by the research associate and principal investigator. The two met to compare findings and achieve consensus by discussion. Data (quotes labeled by theme) were tabulated by theme, faculty or department, professional role, sex, and career stage to identify trends and facilitate interpretation. Themes that emerged from interviews were summarized and grouped according to their inter-relationship in a conceptual framework.

## Results

### Participants

Of 70 individuals invited to participate, 16 either declined or did not respond, and 54 consented (Table 1). While participants expressed some diverse views, described in the following results, there were no clear trends according to sampling characteristics, prior experience with mentorship, or baseline understanding of KT.

**Table 1:** Characteristics of interview participants

Characteristic	Category	Interviewed
		(n, % of 54)
Faculty or Department	Family Medicine	5 (9.3)
	Medicine (internal, emergency)	17 (31.5)
	Nursing	5 (9.3)
	Physical Therapy (occupational, physical, rehabilitation, speech-language pathology)	13 (24.1)
	Surgery (general, orthopaedic)	14 (25.9)
Type of investigator	PhD	26 (48.1)
	Clinician	28 (51.9)
Career stage	Junior	19 (35.2)
	Mid-career	23 (42.6)
	Later career	12 (22.2)
Sex	Female	21 (38.9)
	Male	33 (61.1)

### Current and desired KT knowledge and practice

Most participants had a basic understanding of KT even if they were not familiar with the label. They recognized that its purpose was to promote the use of innovations, achieve better patient care and improve health outcomes.

Getting research findings out into clinical and or community use – not just known but actually implemented and used (05)

To apply the knowledge you get from research into better patient care (012)

Participants most commonly used meetings and publications to implement innovations, though they recognized that many other strategies could be used (Table 2).

**Table 2:** KT strategies used or identified by participants

Theme	Exemplary Quotes
Meetings (conference, workshop, rounds)	• information is conveyed in a lecture style or classroom setting (030)
	• national and international presentations (038)
	• go to the medical rounds or other conference or workshop (02)
	• conferences, lectures, rounds (017)
Continuing education	• through continuing medical education (027)
	• from a clinical perspective what you do is continuing medical education (010)
	• continuing education programming, large group and small group (051)
Publications	• I think of more traditional KT strategies like journal publications (03)
	• if it gets published then hopefully somebody will look at your paper (010)
	• I’ve never really thought of anything other than publishing (011)
	• part of that is publication (035)
Guidelines	• through instituting guidelines or clinical pathways (027)
	• it might be incorporated into a practice guideline (01)
	• I’m mostly involved in guideline development (014)
	• Implementing new practice guidelines (01)
Policies	• through changing policies (027)
	• if I am meeting with a policy maker then I become aware of their interests and what his constituency is interested in (04)
	• we are regularly in contact with government (018)
Financial incentives	• financial incentives to do or financial disincentives not to do something (022)
Facilitators	• you have to find a local champion…to adjust it for their local setting (02)
	• having influential people…advocate and or promote it (035)
	• knowledge brokers…at the point of care facilitating best practice (016)
	• get local opinion leaders involved (054)
Educational outreach/ Academic detailing	• academic detailing…a visitor goes to a practice and works with the individual to identify what their current practice is (033)
	• academic detailing – a resource within your field who can come and observe and inform your practice through an interactive process (052)
	• someone makes an appointment at your office and talks to you about best clinical practice based on research evidence (021)
Internet	• online web-based programs (027)
	• posted on our website (038)
	• web-based teaching resources, repositories of information (026)
Mass media	• we’ve done a lot of television, radio, and web interviews (015)
	• I use the media when possible (05)
	• I do over a hundred media interviews a year (018)
Interaction with users	• sometimes after the information has been developed it goes back to practitioners for some discussion about how they could use it (019)
	• involving them from the start, networking with them (05)
	• engaging the end-users in the design and the conduct of the research (021)
	• debriefing sessions with participants of the research (030)

Several participants noted that they did not practice KT or did not practice it well, either due to the belief that it was not their responsibility, or lack of knowledge and skill.

I don’t see it as my primary role, probably because I’m not comfortable with it (03)

The best thing would be to hire someone who had knowledge and appropriate skills and they could take responsibility (021)

However, most participants were interested in developing the ability to more effectively practice KT. This included knowing when and how to engage in KT, but also how to target particular types of end users, and how to use information systems for doing so.

What KT strategies have been found to be most effective and when is it appropriate to actually engage in KT (030)

How to get research messages across to people in general practice (047)

How can we use new technologies to help (046)

### Preferences for mechanisms of learning about KT

Participants expressed interest in learning about KT through one or more of three broad options, each emphasizing the need for brevity and time management. A few participants said that summary documents would provide quick information about effective KT strategies.

A fact sheet of two pages - then I don't have to read through piles of stuff (04)

Others suggested learning about KT through meetings, underscoring the value of interaction with colleagues while also making efficient use of time.

A workshop would be the most effective – everybody’s too busy to be reading and looking things up (015)

Several participants said that interaction with, and advice from a KT expert would be valuable.

Interacting with individuals who have knowledge of knowledge translation (049)

Having someone who is knowledgeable about it to be able to act as a resource (054)

### Views about KT mentorship

Most participants had some prior experience with clinical or academic mentorship. With respect to KT mentorship, many participants emphasized the value of a mentor over other forms of learning about KT. In part this was due to personal interaction.

There is a difference between interacting with a knowledgeable experienced individual and reading something in a textbook – it’s the quality of the interaction (035)

There’s a greater opportunity to address an individual need when you create a relationship with your mentor which you wouldn’t get in a workshop (041)

Others valued a mentor over other forms of learning about KT because it was a time-efficient means of accessing information that was both credible and tailored to individual needs.

You don’t have to go through a process of judging the information source (051)

They can tailor the information to meet your particular need (038)

Furthermore, KT mentors were seen as individuals that could be engaged on an on-going or as-needed basis in the trajectory of a project to provide feedback or advice. They could also function as linking agents by identifying and connecting with target end-users.

Identifying who are all the relevant knowledge users who would be stakeholders and then actually building connections with them (07)

### Recommendations for KT mentorship program design

Various options for the structure, delivery, and content of a KT mentorship program were articulated. For example, with respect to mentoring model, several participants recommended traditional one-on-one mentorship whereas others said that several individuals could be mentored concurrently so that mentees could learn from each other.

Someone who’s available to provide one-on-one guidance (021)

I could see having a group mentor program because we could learn from each other (019)

Multiple mentors might be necessary, either concurrently or consecutively depending on the needs of a given project.

You might have a mentor for a particular project and when that project folds you seek out another mentor (01)

I probably need someone to look at my specific projects and say "oh, you should be talking to this person or did you ever think of looking up this agency" (03)

Views diverged about the optimal frequency and duration of mentorship. Some participants said that mentoring should occur regularly on a weekly, monthly or yearly basis, or even longer while others thought that interaction would depend on project needs.

It’s a longitudinal process – perhaps five to ten years (013)

It would kind of wax and wane with projects at different stages (03)

Views also differed about in-person versus remote mentorship, with advantages featured in both.

My preference would be face-to-face. You can develop greater rapport than over the telephone, and have a greater chance of building trust and really having meaningful communication where you can really convey what your struggles are (07)

It would be nice to have email access to that person – if it’s a quick question you don't want to wait a whole week to be able to move forward (020)

### Considerations for establishing a KT mentorship program

Four key themes emerged with respect to the considerations and conditions for establishing a KT mentorship program.

### Fostering a culture of mentorship

The majority of recommendations for establishing KT mentorship were related to developing a culture that valued it, primarily through incentives such as recognition for KT activities. In such a culture, KT mentorship would be integrated with education, both training and continuing, and would be naturally propagated.

I'm never evaluated on my KT abilities or whether anything I've done has been translated to users - if these incentives were built in I think this could be very sustainable (011)

Mentorship needs to be valued much more than it is at present – once you attach value to it then people will seek to do it (01)

It should be introduced to people in residency and should be part of faculty development (013)

Create a culture whereby people who received mentorship might evolve into the mentors themselves (054)

Leadership for KT mentorship

Leadership was needed to steward and oversee a KT mentorship program. Participants said that the Ministry of Health, university faculties or departments, hospitals and hospital research institutes, or professional societies, or a collaborative venture should be responsible

There has to be someone at the helm overseeing it (024)

Some sort of collaborative effort between university departments, funding agencies, relevant policy makers, and other stakeholders (038)

Infrastructure for KT mentorship

Funding was needed to compensate mentors, and support staff and various operational activities.

To advertise the services, put on education sessions (020)

Develop a catalogue of available mentors (051)

Establish a mechanism by which individuals could link to each other (049)

A program that facilitated those administrative functions so that the mentor and mentee can concentrate on transfer of expertise (014)

It was also suggested that the program offer guidance for the processes of mentorship to help both mentors and mentees establish functional mentoring relationships.

Protocols for how a mentor and mentee develop shared goals, standard tools and procedures, and guidance for how to enable these mentoring relationships (031)

KT mentors

Most participants noted that a cadre of KT mentors would be needed. A few said that KT mentors should be embedded in organizations as a centralized resource.

They would be another type of support, like having statistical support (020)

Views were mixed about whether mentors should be KT experts or individuals with similar professional training to the mentee. Participants said that identifying good mentors and, in particular, those with expertise in KT would be a challenge.

Somebody who has a lot of experience and knows the literature and is actively involved in KT work (05)

They understand what I do…somebody who has more or less the same job description or experience (023)

### Conceptual framework

**Fig. 1:**
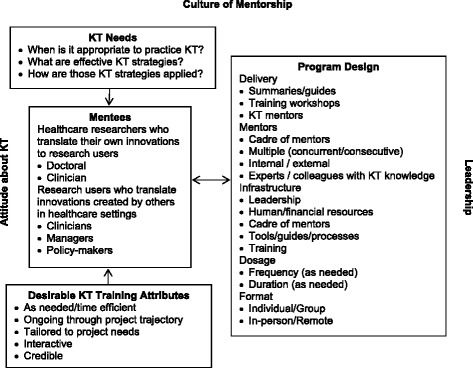
Conceptual framework of KT mentorship design and determinants

Figure 1 displays a visual conceptual framework of the inter-relationship of themes that emerged from interviews. Mentees, both researchers and research users, may be influenced by their KT informational needs and their preferences for the attributes of KT training, and may benefit from KT mentorship programs that address these needs and preferences. Participation in KT mentorship may be influenced by mentee interest in KT, and attitude about whether they are responsible for KT. The design of KT mentorship programs that can address these needs and preferences is complex, and would require considerable infrastructure. Strong and dedicated leadership may influence the operationalization and sustainability of such a program. Overall, a culture must be established that promotes value for KT mentorship, and incentivizes KT mentorship through recognition for the time and effort dedicated to its practice, and its impact.

## Discussion

The purpose of this study was to explore how mentoring could be used to help researchers and research users implement evidence-based healthcare innovations. Most participants were familiar with the concept of KT but said they lacked expertise and were interested in developing KT capacity. Mentorship offered several advantages over other forms of learning about KT because it would provide credible, tailored information on an on-going and as-needed basis. While views about mentoring program design varied, there were no trends by organizational affiliation, specialty, sex, career stage, or researcher versus research user, so a range of options may be required to suit different needs. Findings, including KT needs, preferences for the attributes of KT training, and insight on the infrastructure and overall conditions needed to deliver KT mentorship programs were summarized in a conceptual framework that could be used by others to guide the development of evaluation of KT mentoring programs.

Application of these findings may be limited by transferability or relevance of the findings to other settings. We attempted to mitigate this through purposive sampling of participants based on characteristics that may have influenced their views. While we achieved thematic saturation and found no trends according to sampling characteristics, we sampled only from two faculties and three departments at one university, and some specialties may be under-represented. Further research is needed to confirm whether these findings are true of researchers or research users in other settings. Still, according to our recent literature search, this was and remains the first and only study to explore how mentorship can be used to develop KT capacity, and the findings provide direction for policy, practice and ongoing research. To address what was referred to as a paucity of KT educational and professional development opportunities, another study published in 2013 described the development of a KT community of practice that shared information through a blog, virtual seminar series, and quarterly newsletter [35]. This initiative also included a peer mentorship component involving events during which senior members shared experiences with junior members. Thus our study, which explored needs, preferences and the capacity to support KT mentorship remains unique. Though our interviews took place in 2009, a survey of 1,071 researchers and researcher users published in 2014 found that more than 85 % were interested in learning more about KT, and of those 47 % said they required beginner level training, thus the need for KT training remains relevant [36].

First, given that KT training programs may be few or may not include mentorship, organizational infrastructure for offering KT mentorship more broadly is needed. This includes leadership from one or more organizations to assume the responsibility for organizing and supporting KT mentorship. A key function of such an organization or collaborative effort would be to establish a culture that valued and was receptive to mentorship, and provided the necessary resources. The lack of institutional resources for mentoring programs, and the need to foster a culture of mentorship have also been identified by others as barriers to mentorship [37]. Guidance on how to assess and change organizational culture is available to support such a shift [38–40]. The shift would not be immediate, and may take place over three broad phases including preparing for, adopting and then routinizing the use of mentorship to develop KT capacity [40]. Its appears that many healthcare organizations are moving in this direction by identifying local leaders, creating dedicated units and nurturing a culture that embraces quality improvement, and may value information on how mentorship can support these efforts [41, 42].

The need to identify and assemble a cadre of skilled mentors with expertise in KT was recognized as a challenge by participants of this research. Other research has revealed difficulty in finding mentors and establishing productive relationships, and identified the need for training of both mentors and mentees [43–46]. In this study the need for more than one KT mentor was identified. Some noted that mentors often served as linking agents to other mentors, which has been identified in other research [21]. Other research involving interviews with 100 clinician investigators and 28 of their mentors also revealed that a single mentor could not fulfill the diverse mentoring requirements [47]. While views were mixed about whether mentors should be KT experts or individuals with similar professional alignment, several participants recommended that KT experts or scientists be embedded in organizations to provide mentorship. In this model the KT expert might work with individual researchers, clinicians, or managers, or teams to offer training and support for the implementation of innovations.

With respect to program design there must be flexibility in format, delivery and content to suit various needs. Participants underscored the need for access to quick, concise resources about KT. This would perhaps suffice for many who expressed a need to know which KT strategies were most effective. However, many participants expressed interest in tailored, personal advice on an ongoing or as-needed basis to address specific issues, suggesting the need for more formal or ongoing mentorship. In the United States the Society of Hospital Medicine offered conferences and detailed toolkits to support the implementation of innovations in eight clinical topics but found that improvements were modest and health professional teams were frustrated [48]. Following subsequent delivery of a program in which hospitalists with expertise in both KT and relevant clinical matter mentored hospitalists in more than 300 teams through teleconferences, group webinars, site visits and two-day intensive training sessions, preliminary data showed significant improvement in patient outcomes. This study highlights the value of mentorship compared with other mechanisms for sharing KT information, and suggests that mentors need not be embedded in organizations, but could provide mentoring to internal champions.

Ongoing research is needed on several fronts. Concepts articulated by participants and captured in the conceptual framework suggest that the development and evaluation of KT mentorship could be guided by social constructivist learning theory which proposes that, although the learner is independent and self-regulated, learning is a result of interaction with the environment so it is socially mediated by others and by the over-riding culture [49]. The PARIHS framework may also be relevant because it specifies that successful application of knowledge is determined by the characteristics of the knowledge, contextual environment including leadership, culture and receptivity, and facilitation [50]. Further research is needed to assess which of these and other theories best support the development and/or evaluation of KT mentorship. A systematic review of academic mentorship revealed that the dyad model was most common, and the formation of mentor-mentee pairs was the most evaluated aspect in eligible studies [51]. Therefore, further research is needed to investigate the benefits and implications of alternative models for KT mentorship. This would include an embedded KT expert that could work with individuals or teams, and mentoring of individuals or teams by an external KT mentor. Further research is also needed to establish the core curriculum of a training program specific to KT, and how it could best be reinforced with mentoring. Valuable insight could be gained through evaluations of existing KT training initiatives [10–13, 48]. Organizations could then use this guidance to establish programs for developing KT capacity. Upon conclusion of this study we held a one-day meeting to share the findings with participants and other clinicians, managers and KT researchers. In ongoing research we could evaluate whether and how those departments implemented mentorship programs. We are also currently using the findings to develop an international KT mentorship program to provide support for those who develop and implementation guidelines therefore we will be able to further develop the conceptual framework.

## Conclusions

This is the first study to explore the optimal design of mentorship programs to instill KT knowledge and skills among researchers and research users. The design of KT mentorship programs that can address the needs and preferences articulated by participants is complex, and would require considerable infrastructure. Individual interest in, and attitude about KT, leadership support, and an overall culture that values and incentivizes KT mentorship may influence the development, impact and sustainability of such programs. These concepts were captured in a framework that can be used by others to design or evaluate KT mentorship programs.

## Additional files

Additional file 1:
**Relevance, Appropriateness, Transparency and Soundness (RATS) principles for the reporting of qualitative research.**
Additional file 2:
**KT Mentor Interview Guide.**
