# Supplementary material for: Designing a knowledge translation mentorship program to support the implementation of evidence-based innovations
Source: BMC Health Serv Res. 2015 May 14;15:198. doi: 10.1186/s12913-015-0863-7 (PMC4443629; doi:10.1186/s12913-015-0863-7)
Supplement: Additional file 1: — Relevance, Appropriateness, Transparency and Soundness (RATS) principles for the reporting of qualitative research. [file 12913_2015_863_MOESM1_ESM.doc]

**Additional file 1**

**Relevance, Appropriateness, Transparency and Soundness (RATS)
principles for the reporting of qualitative research**

Clark JP: How to peer review a qualitative manuscript. In Peer Review in Health Sciences. Second edition. Edited by Godlee F, Jefferson T. London: BMJ Books; 2003:219-235

**R – relevance of study question**

- Research question explicitly stated
- Research question justified and linked to the existing knowledge base (empirical research, theory, policy)

**A – appropriateness of qualitative method**

- Study design described and justified i.e., why was a particular method (e.g., interviews) chosen?

**T – transparency of procedures**

- Criteria for selecting the study sample justified and explained
- Details of how recruitment was conducted and by whom
- Details of who chose not to participate and why
- Data collection method outlined
- Study group and setting clearly described
- End of data collection justified and described
- Do the researchers occupy dual roles (clinician and researcher)? Are the ethics of this discussed?
- Ethics approval cited
- Informed consent process explicitly and clearly detailed

**S – soundness of interpretive approach**

- Analytic approach described in depth and justified: Description of how themes were derived from the data (inductive or deductive); Evidence of alternative explanations being sought; Analysis and presentation of negative or deviant cases
- Description of the basis on which quotes were chosen: Semi-quantification when appropriate; Illumination of context and/or meaning, richly detailed
- Method of reliability check described and justified: e.g., was an audit trail, triangulation, or member checking employed? Did an independent analyst review data and contest themes? How were disagreements resolved?
- Findings presented with reference to existing theoretical and empirical literature, and how they contribute
- Strengths and limitations explicitly described and discussed
- Detail of methods or additional quotes contained in appendix
