# Supplementary material for: Designing a knowledge translation mentorship program to support the implementation of evidence-based innovations
Source: BMC Health Serv Res. 2015 May 14;15:198. doi: 10.1186/s12913-015-0863-7 (PMC4443629; doi:10.1186/s12913-015-0863-7)
Supplement: Additional file 2: — KT Mentor Interview Guide. [file 12913_2015_863_MOESM2_ESM.doc]

OPENING REMARKS

Thank you for agreeing to participate in this study. The interview will require approximately 30 minutes. I will be asking you several questions about your understanding and use of knowledge translation, and your thoughts on how mentorship could support knowledge translation. Our conversation is being recorded, but your responses will remain confidential. Before we begin, do you have any questions?

PROFESSIONAL ROLE/CAREER STAGE

Would you describe yourself as a clinician, researcher, manager, or educator?

Would you describe yourself as junior, mid career or late career?

UNDERSTANDING OF KT

What does knowledge translation (which I will call KT for short) mean to you?

Review with all:

*Promoting use of research in practice / encourage evidence informed decision making*

*Includes continuing education, quality improvement or other strategies that improve quality of care*

*Involves tailoring evidence and interventions/strategies to user needs/constraints*

USE OF KT

What KT methods or strategies do you use, or are you familiar with?

KT NEEDS

What more would you like to know about KT?

How, or in what format would you prefer to receive information about KT? Why this preference?

MENTORSHIP

Briefly describe your experience with receiving or providing mentorship.

KT MENTORSHIP

We are considering whether and how guidance from a mentor could support KT.

Would you be interested in receiving some form of KT mentorship?

If NO

Enquire about reason, and go to KT MENTORSHIP SUPPORT

If YES

What is your preference for format of KT mentoring? Why this preference?

KT MENTORSHIP IMPACT

What would KT mentorship achieve over reading about KT or going to a workshop about KT?

KT MENTORSHIP SUPPORT

What do you believe are desirable characteristics of a KT mentor?

What are the essential components or services of a KT mentorship program?

What infrastructure or resources are required to offer such program?

Who should be responsible for sponsoring/offering/arranging a KT mentorship program?

What challenges might arise in offering or participating in a KT mentorship program?

CONCLUSION

Do you have any other suggestions for supporting KT mentorship?

Thank you for taking the time to speak with me.
